# Deleterious mutation V369M in the mouse *GCGR* gene causes abnormal plasma amino acid levels indicative of a possible liver–α-cell axis

**DOI:** 10.1042/BSR20210758

**Published:** 2021-06-02

**Authors:** Qiaofeng Liu, Guangyao Lin, Yan Chen, Wenbo Feng, Yingna Xu, Jianjun Lyu, Dehua Yang, Ming-Wei Wang

**Affiliations:** 1 School of Pharmacy, Fudan University, Shanghai 201203, China; 2 School of Life Science and Technology, ShanghaiTech University, Shanghai 201210, China; 3 Department of Pathology, InnoStar BioTech Nantong Co., Ltd., Nantong 226133, China; 4 The National Center for Drug Screening and CAS Key Laboratory of Receptor Research, Shanghai Institute of Materia Medica, Chinese Academy of Sciences (CAS), Shanghai 201203, China; 5 School of Basic Medical Sciences, Fudan University, Shanghai 200032, China

**Keywords:** glucagon receptor, mouse model, mutation, proliferation, V369M

## Abstract

Glucagon plays an important role in glucose homeostasis and amino acid metabolism. It regulates plasma amino acid levels which in turn modulate glucagon secretion from the pancreatic α-cell, thereby establishing a liver–α-cell axis described recently. We reported previously that the knock-in mice bearing homozygous V369M substitution (equivalent to a naturally occurring mutation V368M in the human glucagon receptor, GCGR) led to hypoglycemia with improved glucose tolerance. They also exhibited hyperglucagonemia, pancreas enlargement and α-cell hyperplasia. Here, we investigated the effect of V369M/V368M mutation on glucagon-mediated amino acid metabolism. It was found that *Gcgr*^V369M+/+^ mice displayed increased plasma amino acid levels in general, but significant accumulation of the ketogenic/glucogenic amino acids was observed in animals fed with a high-fat diet (HFD), resulting in deleterious metabolic consequence characteristic of α-cell proliferation and hyperglucagonemia.

## Introduction

Glucagon, a peptide hormone secreted by the pancreatic islet α-cell, regulates carbohydrate homeostasis and lipid metabolism through its cognate glucagon receptor (GCGR) [1–3]. It releases glucose from the liver by glycogenolysis and stimulates hepatic amino acid uptake [3–5]. It also promotes hepatic amino acids turnover by induction of enzymatic activities via the urea cycle [5,6]. *Gcgr* knockout or administration of GCGR-blocking antibodies affected serum amino acid availability [7–11]. Circulating amino acids could act as a nutrient sensing circuit between the liver and the pancreas to control glucagon secretion from the α-cell, thereby forming a feedback loop, i.e., liver–α-cell axis [7,8,12–16].

Mahvash disease, an autosomal recessive disorder caused by biallelic inactivating GCGR mutations, is rare but has been gradually recognized [17,18]. It manifests itself as hyperglucagonemia without glucagonoma syndrome, hyperaminoacidemia, α-cell proliferation and hereditary pancreatic neuroendocrine tumor (PNET) [17–21]. We reported previously that a naturally occurring mutation V368M (equivalent to mouse V369M) in the human GCGR led to reduced ligand binding and down-regulation of glucagon signaling [22]. *Gcgr*^V369M+/+^ mice displayed lower fasting blood glucose levels with improved glucose tolerance compared with the wildtype (WT) controls. They also exhibited hyperglucagonemia, pancreas enlargement and α-cell hyperplasia [22].

The current study was designed to investigate the effect of V368M/V369M mutation on amino acid metabolism under normal and high-fat diet (HFD) conditions using *Gcgr*^V369M+/+^ mice. Apart from plasma abnormal amino acid levels, marked accumulation of the ketogenic/glucogenic amino acids was noted in HFD-fed animals accompanied by hyperglucagonemia and α-cell hyperplasia.

## Experimental procedures

### Animals and diet

Generation of *Gcgr*^V369M+/+^ mice was described previously [22]. The genotypes of all the mice were verified by PCR and confirmed by DNA sequencing (Supplementary Figure S1). All animal models were on a C57BL/6J background and housed in a specific-pathogen free (SPF) animal facility at the vivarium of Shanghai Institute of Materia Medica, Chinese Academy of Sciences. The male mice were used for all indicated studies and the offspring of WT littermates were used as controls for *Gcgr*^V369M+/+^ mice. Mice were fed a control standard chow diet (SCD, Supplementary Figure S2A) or a HFD (Supplementary Figure S2B), watered *ad libitum* under a 12-h light–dark cycle (lights on from 8:00 a.m. to 8:00 p.m.) at constant temperature (22°C). Euthanasia was carried out under carbon dioxide asphyxia at the end of the present study. All animal experiments were approved by the Animal Care and Use Committee, Shanghai Institute of Materia Medica, Chinese Academy of Sciences (IACUC number: 2020-02-WMW-10).

### Amino acid analysis

The amino acid analysis of physiological samples refers to the TRAQ™ reagent application kit protocol (AB Sciex, Framingham, MA, U.S.A.). In short, the mixture of 40 μl samples and 10 µl sulfosalicylic acid was centrifuged at 10000×*g* for 2 min. Ten microliters of the supernatant was then transferred with 40 μl labeling buffer and mixed by vortex. After spinning, 10 μl diluted supernatant was mixed with 5 μl diluted TRAQ™ reagent Δ8 (pre-labeling sample) followed by incubation at room temperature (RT) for at least 30 min. After that, 5 μl hydroxylamine was added to the mixture. The samples were completely dried in a centrifugal vacuum concentrator (generally not more than 1 h) and 32 μl reconstituted amino acid internal standard solution was added to each dried TRAQ™ reagent Δ8-labeled sample for centrifugation and detection by 4000 QTRAP LC/MS/MS (Sciex).

### Histology

The mouse pancreases and livers were collected and subsequently fixed in 4% paraformaldehyde (PFA). The samples were then embedded in paraffin and sliced into 4-μm cross-sections. One section per pancreas or liver was stained with Hematoxylin and Eosin (H&E) as previously described [23]. Processed sections were evaluated by physician or scientist with extensive experience in pancreas and liver histology for degree of lesion, number of lesions and description of abnormalities observed. Pathological diagnosis was given as minor lesion (+), mild lesion (++), moderate lesion (+++) and severe lesion (++++).

### Immunofluorescence

Prior to immunostaining, the sections were treated sequentially with 3% hydrogen peroxide, citrate-based antigen retrieval solution (Beyotime Biotechnology, Shanghai, China) and goat serum blocking dilution. Insulin and glucagon were stained with anti-insulin (1:200; Cell Signaling Technology, Danvers, MA, U.S.A.) and anti-glucagon (1:200; Abcam, Cambridge, MA, U.S.A.) antibodies, respectively, followed by reacting with 1:1000 either Alexa Fluor 488-conjugated donkey anti-rabbit or Alexa Fluor 594-conjugated donkey anti-mouse IgG antibodies (Invitrogen, Carlsbad, CA, U.S.A.), while nuclei were visualized with 4′,6-diamidino-2-phenylidole (DAPI; Invitrogen). Sections were analyzed using a Vectra automated quantitative pathology system (PerkinElmer, Waltham, MA, U.S.A. ).

### Blood chemistry

At the end of the study, blood samples were collected form the inner canthus vein plexus under anesthesia by intraperitoneal injection of 1% (w/v) pentobarbital sodium (70 mg/kg) and stored at −80°C. Plasma insulin, glucagon and glucagon-like peptide-1 (GLP-1) levels were measured using ELISA kits from Crystal Chem (Chicago, IL, U.S.A.). Plasma albumin (ALB), blood urea nitrogen (BUN) and creatinine (CRE) were analyzed by a JCA-BM6010/C analyzer (JEOL Ltd., Tokyo, Japan).

### Statistical analysis

Statistical analyses were performed using Prism 8 software (GraphPad, San Diego, CA, U.S.A.) and amino acid clustering were analyzed by MetaboAnalyst 5.0 (https://www.metaboanalyst.ca/MetaboAnalyst/home.xhtml). Statistical significance was determined using two-tailed Student’s *t* test. A threshold of *P*<0.05 is considered to be statistically significant. Data shown are means ± SEM from multiple experiments performed in duplicate.

## Results

### Plasma amino acid level

**Figure 1:**
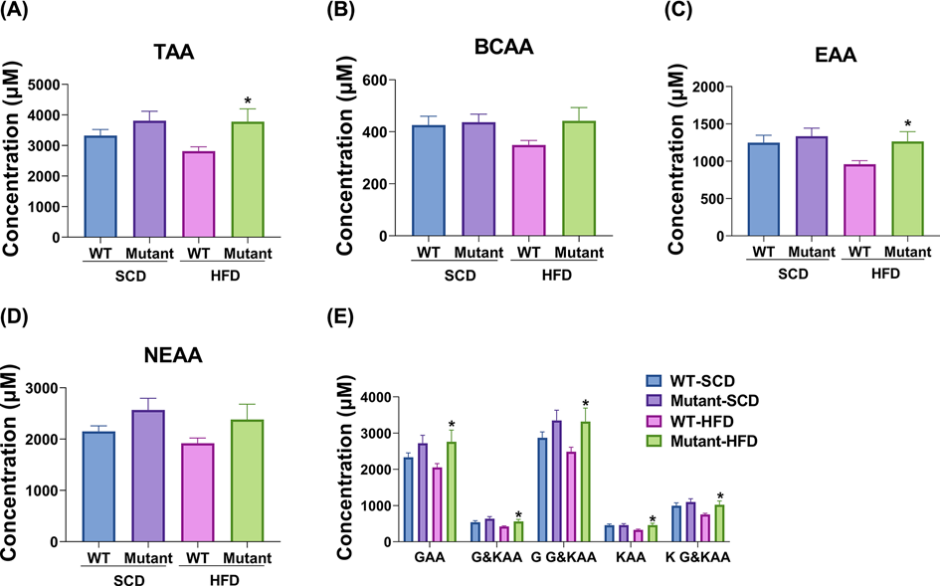
Evaluation of amino acid levels in *Gcgr^V369M+/+^* (mutant) and WT mice Plasma amino acid concentrations were measured in 35–39 weeks old male mice fed with SCD or HFD for 23–35 weeks. (**A**) TAA (the sum of all α amino acids examined). (**B**) BCAA (**C**) EAA (**D**) NEAA (**E**) GAA, G&KAA, G G&KAA, KAA and K G&KAA. Data shown are means ± SEM, *P<0.05 using Student’s *t* test, *n*=15–17.

As shown in Figure 1, the plasma concentrations of total α-amino acids (TAA, Figure 1A), branched-chain amino acids (BCAA, Figure 1B), essential amino acids (EAA, Figure 1C) and nonessential amino acids (NEAA, Figure 1D) in *Gcgr*^V369M+/+^ mice were not significantly different from that of WT mice fed with SCD. *Gcgr*^V369M+/+^ mice receiving HFD showed statistically significant increases in TAA (Figure 1A), EAA (Figure 1C), glucogenic amino acids (GAA), glucogenic and ketogenic amino acids (G&KAA), the summation of GAA and G&KAA (G G&KAA), ketogenic amino acids (KAA) and the summation of KAA and G&KAA (K G&KAA) (Figure 1E) levels compared with the WT.

**Figure 2:**
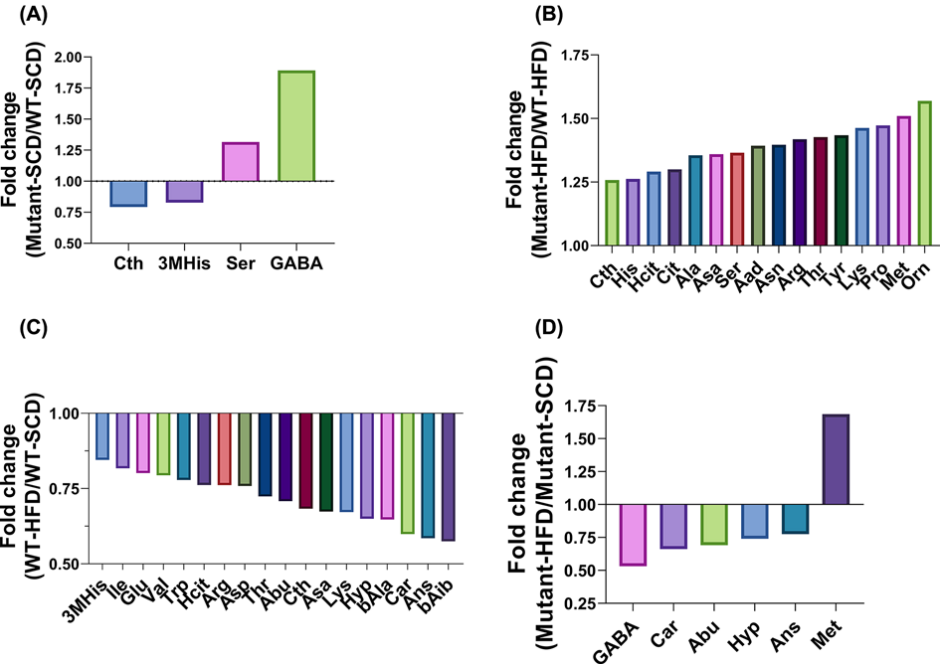
Fold change of plasma amino acid levels (**A**) *Gcgr*^V369M+/+^ (mutant) mice displayed significantly different amino acids levels compared with WT mice fed with SCD. (**B**) Sixteen amino acids were significantly increased in *Gcgr*^V369M+/+^ mice in HFD group compared with WT mice. (**C**) Eighteen amino acids were significantly decreased in WT mice fed with HFD compared with WT mice fed with SCD. (**D**) Amino acid levels were significantly changed in *Gcgr*^V369M+/+^ mice fed with HFD compared with *Gcgr*^V369M+/+^ mice fed with SCD. Plasma amino acid concentrations were measured in 35–39 weeks old male mice fed with SCD or HFD for 23–35 weeks. *P*<0.05 using Student’s *t* test, *n*=15-17.

Specifically, 42 types of amino acids were examined and the results show that among *Gcgr*^V369M+/+^ mice fed with SCD, there was a significant decline in plasma Cth (−20.75%) and 3MHis (−17.14%) concentrations along with marked accumulation of Ser (+31.59%) and GABA (+89.16%) compared with the WT mice (Table 1 and Figure 2A). In contrast, when on an HFD, *Gcgr*^V369M+/+^ mice exhibited higher Cth (+25.79%), His (+26.23%), Hcit (+29.12%), Cit (+30.0%), Ala (+35.52%), Asa (+35.94%), Ser (+36.53%), Aad (+39.28%), Asn (+39.67%), Arg (+41.82%), Thr (+42.65%), Tyr (+43.41%), Lys (+46.26%), Pro (+47.29%), Met (+50.98%) and Orn (+56.91%) levels than that of the WT (Table 1 and Figure 2B). It was noted that a number of amino acids were decreased in WT mice receiving HFD, such as 3MHis (−15.49%), Ile (−18.28%), Glu (−19.85%), Val (−20.62%), Trp (−22.14%), Hcit (−23.83%), Arg (−23.84%), Asp (−24.13%), Thr (−27.60%), Abu (−29.18%), Cth (−31.67%), Asa (−32.64%), Lys (−32.82%), Hyp (−35.02%), bAla (−35.29%), Car (−40.11%), Ans (−41.47%) and bAib (−42.52%), compared with SCD controls (Table 1 and Figure 2C). However, only Met level was increased (+68.56%) whereas that of Ans (−22.41%), Hyp (−25.90%), Abu (−30.82%), Car (−33.80%) and GABA (−46.79%) were decreased in *Gcgr*^V369M+/+^ mice fed with HFD as opposed to the counterparts consuming SCD (Table 1 and Figure 2D). After excluding the effect of different diets on amino acid levels, we found that GCGR bearing V369M may lead to elevated levels of Asp, Trp, bAla, Hyp, Car, Ser, Glu, Hcit, bAib, Ans, Thr, Pro, Val, Lys, Cit, Asa, Ile, Ala, Arg, Aad, Orn and Met.

**Table 1:** Plasma amino acid concentrations (**μ**M)

	SCD		HFD	
	WT	*Gcgr*^V369M+/+^	WT	*Gcgr*^V369M+/+^
1MHis	6.10 ± 0.40	7.05 ± 0.60	5.36 ± 0.39	6.83 ± 0.93
3MHis	8.93 ± 0.40	7.40 ± 0.60*	7.55 ± 0.49^#^	8.69 ± 0.76
Aad	9.98 ± 1.06	9.01 ± 0.72	7.63 ± 0.48	10.63 ± 0.90**
Abu	7.65 ± 0.47	8.16 ± 0.68	5.41 ± 0.49^##^	5.64 ± 0.43^##^
Ala	402.86 ± 27.62	456.85 ± 33.54	349.37 ± 20.24	473.47 ± 46.08*
Ans	2.88 ± 0.19	2.56 ± 0.17	1.68 ± 0.14^##^	1.98 ± 0.18^#^
Arg	119.91 ± 10.05	120.12 ± 7.88	91.31 ± 6.53^#^	129.53 ± 15.84*
Asa	0.56 ± 0.06	0.49 ± 0.04	0.38 ± 0.04^#^	0.51 ± 0.05*
Asn	52.92 ± 3.36	73.69 ± 10.82	46.23 ± 2.00	64.57 ± 6.69**
Asp	17.61 ± 0.62	18.11 ± 1.97	13.36 ± 1.48^#^	13.89 ± 1.14
bAib	0.12 ± 0.02	0.12 ± 0.02	0.07 ± 0.01^#^	0.09 ± 0.02
bAla	30.04 ± 3.97	28.72 ± 4.04	19.44 ± 3.00^#^	19.00 ± 3.46
Car	1.42 ± 0.08	1.30 ± 0.08	0.85 ± 0.11^##^	0.86 ± 0.08^##^
Cit	67.78 ± 5.53	72.29 ± 5.82	55.67 ± 2.66	72.37 ± 6.85*
Cth	1.18 ± 0.07	0.93 ± 0.05**	0.80 ± 0.08^##^	1.01 ± 0.06*
Cys	35.49 ± 4.06	31.81 ± 4.94	29.14 ± 4.13	34.75 ± 7.00
EtN	12.24 ± 0.85	13.56 ± 1.20	13.08 ± 1.29	15.91 ± 1.41
GABA	0.87 ± 0.09	1.64 ± 0 .25**	2.74 ± 1.66	0.87 ± 0.22^#^
Gln	714.57 ± 32.53	869.25 ± 80.21	684.70 ± 40.47	831.02 ± 80.13
Glu	40.91 ± 1.82	39.76 ± 3.12	32.79 ± 3.20^#^	35.37 ± 2.43
Gly	367.54 ± 30.75	428.48 ± 42.33	320.93 ± 38.21	507.76 ± 152.24
Hcit	0.82 ± 0.05	0.91 ± 0.07	0.62 ± 0.04^##^	0.81 ± 0.07*
Hcy	0.02 ± 0.00	0.02 ± 0.00	0.01 ± 0.00	0.02 ± 0.00
His	74.97 ± 4.50	94.53 ± 10.76	66.14 ± 3.58	83.49 ± 7.91*
Hyl	0.53 ± 0.08	0.57 ± 0.08	0.40 ± 0.05	0.60 ± 0.13
Hyp	16.76 ± 1.20	18.80 ± 1.45	10.89 ± 0.78^##^	13.93 ± 1.71^#^
Ile	81.35 ± 5.95	81.02 ± 5.35	66.48 ± 3.28^#^	83.11 ± 9.26
Leu	115.94 ± 8.90	119.87 ± 9.31	101.56 ± 5.51	126.35 ± 15.10
Lys	338.47 ± 29.15	338.93 ± 28.78	227.35 ± 13.59^##^	332.56 ± 37.78*
Met	27.41 ± 1.66	25.51 ± 2.93	28.48 ± 3.23	43.00 ± 6.26*^#^
Orn	57.66 ± 5.28	78.90 ± 12.60	52.84 ± 3.78	82.91 ± 12.70*
PEtN	12.09 ± 0.55	10.51 ± 0.57	9.80 ± 1.07	10.95 ± 1.00
Phe	68.71 ± 5.15	83.19 ± 10.89	58.60 ± 3.12	71.99 ± 7.33
Pro	99.89 ± 6.77	126.47 ± 13.67	83.44 ± 4.84	122.87 ± 16.16*
PSer	0.05 ± 0.01	0.06 ± 0.01	0.06 ± 0.01	0.08 ± 0.01
Sar	3.87 ± 0.57	3.79 ± 0.57	2.91 ± 0.53	3.55 ± 0.39
Ser	148.85 ± 7.69	195.78 ± 19.42*	136.65 ± 6.74	186.46 ± 16.40**
Tau	553.62 ± 27.31	502.49 ± 22.94	476.35 ± 47.02	542.54 ± 42.36
Thr	234.78 ± 23.66	271.00 ± 23.89	170.05 ± 8.51^#^	242.50 ± 23.38**
Trp	76.96 ± 5.45	85.09 ± 6.73	59.92 ± 3.96^#^	69.16 ± 8.33
Tyr	75.75 ± 5.85	113.65 ± 22.60	64.98 ± 3.46	93.19 ± 10.09**
Val	228.44 ± 19.25	235.70 ± 17.10	181.35 ± 8.84^#^	232.59 ± 26.98

Plasma amino acid concentrations were measured in 35–39 weeks old male mice fed with SCD or HFD for 23–35 weeks. Data shown are means ± SEM, *n*=15–17. *P<0.05 and **P<0.01 using Student’s *t* test and compared with WT mice on the same diet. ^#^*P*<0.05 and ^##^*P*<0.01 using Student’s *t* test and compared with mice of the same genotype fed with SCD.

Twenty α-amino acid types (Thr, His, Lys, Met, Val, Ile, Leu, Phe, Trp, Asn, Ser, Gly, Gln, Asp, Ala, Glu, Arg, Pro, Cys and Tyr) are produced by proteolysis, while 42 amino acid types were detected in our study, more than 50% are intermediates during metabolism. Therefore, we further analyzed the changes of these 20 amino acids. When on an SCD, *Gcgr*^V369M+/+^ mice only had increased Ser level compared with WT control (Supplementary Figure S3A), whereas high levels of His, Ala, Ser, Asn, Arg, Thr, Tyr, Lys, Pro and Met were observed if they received HFD (Supplementary Figure S3B). After excluding the effect of diets, we found that *Gcgr*^V369M+/+^ mice had elevated levels of Asp, Trp, Ser, Glu, Thr, Pro, Val, Lys, Ile, Ala, Arg, Leu, Cys, Gly and Met (Supplementary Figure S3C). Their TAA was also higher than the WT (Figure 1A). More pronounced elevation of some amino acids was reported in *Gcgr*^−/−^ mice accompanied by unchanged levels of some other amino acids [8,12,15]. It is conceivable that the impact of V369M mutation only causes a moderate effect on amino acid metabolism compared with that of GCGR knockout.

### Individual variation

**Figure 3:**
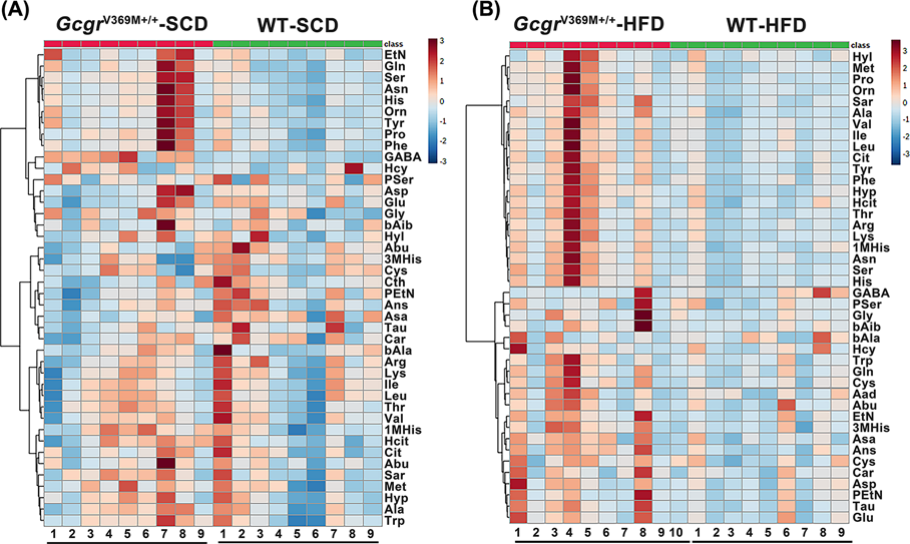
Variation of amino acid levels in *Gcgr*^V369M+/+^ mice Amino acid clustering of the 42 types differentially accumulated in the plasma between *Gcgr*^V369M+/+^ (red) and WT (green) mice fed with SCD (**A**) or HFD (**B**). Scale represents normalized counts using the variance stabilizing transformation (VST). Plasma amino acid concentrations were measured in 39-week-old male mice fed with SCD or HFD for 35 weeks. *n*=9–10.

Clustering of the 42 amino acid types with the highest variance across samples revealed a clear separation between the two mouse genotypes (Figure 3A). Among them, three *Gcgr*^V369M+/+^ mice (numbers 1, 7 and 8) exhibited high levels of Etn, Gln, Ser, Asn, His, Orn, Tyr, Pro, Phe and GABA when fed with SCD versus WT controls. They also manifested mild hepatocyte degeneration, necrosis and steatosis (Supplementary Figure S4). Individual differences thus contributed to the individual variation observed in the present study. However, following HFD, almost every *Gcgr*^V369M+/+^ mouse demonstrated higher amino acid concentrations than that of the WT (Figure 3B).

### Liver histology

**Figure 4:**
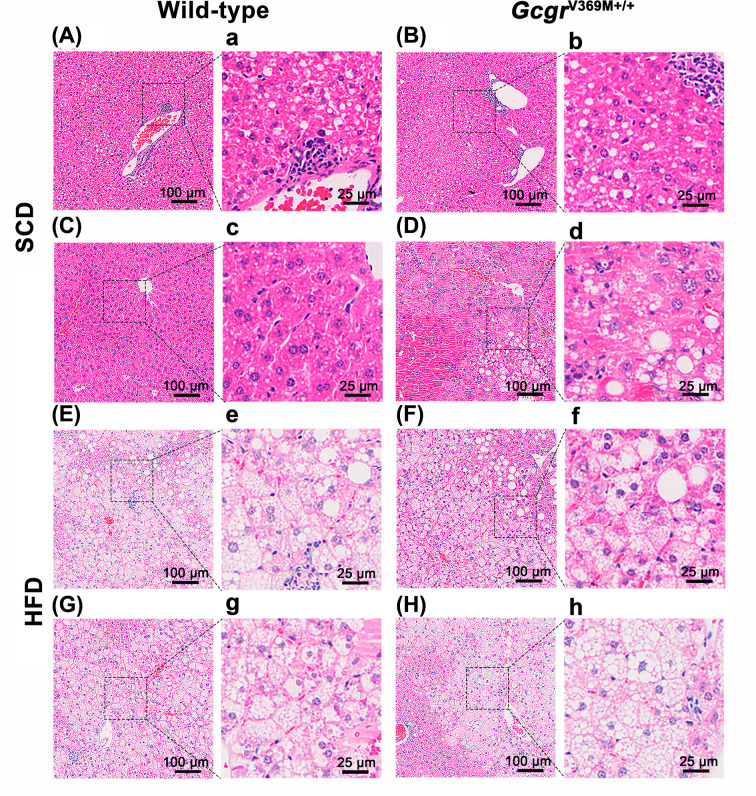
Liver histology of *Gcgr*^V369M+/+^ mice Microscopy evaluation (H&E staining) was conducted on 39-week-old male mice fed with SCD or HFD for 35 weeks. *n*=9–10. Scale bars are indicated in the slides. Images of low (100 μm) and high (25 μm) magnifications from two representative animals of each group are displayed. (**A, a, C, c**) WT mice fed with SCD. (**B, b, D, d**) *Gcgr*^V369M+/+^ mice fed with SCD. (**E, e, G, g**) WT mice fed with HFD. (**F, f, H, h**) *Gcgr*^V369M+/+^ mice fed with HFD.

The liver histology was evaluated and scored (Figure 4 and Table 2). Microscopically, in comparison with WT controls, 3/9 of *Gcgr*^V369M+/+^ mice manifested mild hepatocyte degeneration, necrosis and steatosis, accompanied by oval cells proliferation, hepatocyte nucleus huge, hepatocyte intranuclear inclusions, hyperpigmentation and inflammatory cell infiltration (Figure 4D, d and Supplementary Figure S4). Hepatocellular adenoma was diagnosed only in one animal (data not shown): nodules are proliferative and well-differentiated hepatocytes with steatohepatitis and more than 80% of the liver cells in this tumor are steatosis. At the same time. WT mice on HFD had increased levels of hepatic fat content in histological preparations, presenting with minor to mild inflammatory cell infiltration and moderate to severe fatty infiltration of the liver, compared with mice on SCD. However, there were similar pathological changes between WT and *Gcgr*^V369M+/+^ mice receiving HFD.

**Table 2:** Histological characteristics of the liver

Group			SCD		HFD	
			WT	*Gcgr*^V369M+/+^	WT	*Gcgr*^V369M+/+^
Number			9	9	10	9
Organ	Lesion	Degree	Lesion number			
Liver	Steatosis and cytoplasmic glycogen	+	5	2	1	2
		++	3	3	0	0
	Hepatocyte degeneration/necrosis	++	0	3	0	0
	Inflammatory cell infiltration	+	1	0	7	9
		++	0	3	1	0
	Steatosis	+	0	1	0	0
		++	0	2	0	2
		+++	0	0	3	2
		++++	0	0	6	3
	Oval cells proliferation	++	0	3	0	0
	Hepatocyte nucleus huge	++	0	3	0	0
	Hepatocyte intranuclear inclusions	+	0	1	0	0
		++	0	2	0	0
	Hyperpigmentation	++	0	3	0	0
	Small granuloma	+	3	4	1	2
	Hepatocellular adenoma	✓	0	1	0	0

Liver histology examination was conducted in 39-week-old male mice fed with SCD or and HFD for 35 weeks. *n*=9–10. ‘✓’ abnormal; ‘+’ minor lesion; ‘++’ mild lesion; ‘+++’ moderate lesion; ‘++++’ severe lesions.

### Pancreatic manifestation

**Figure 5:**
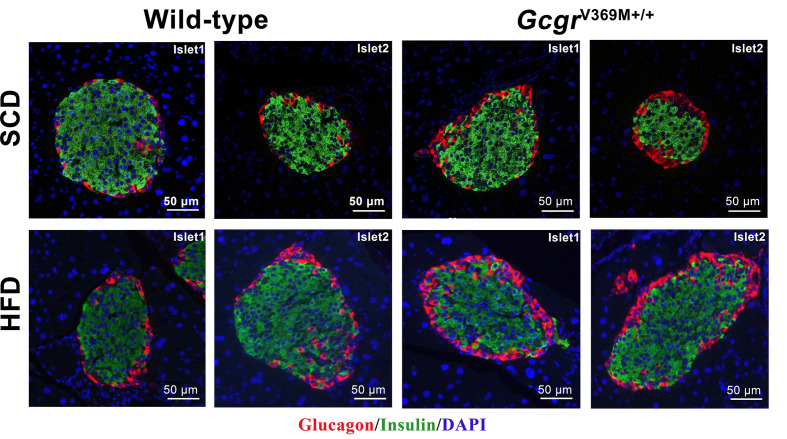
α-cell hyperplasia in *Gcgr*^V369M+/+^ mice Morphology of the pancreatic islets from 39-week-old male WT and *Gcgr*^V369M+/+^ mice fed with SCD or HFD for 35 weeks. Immunofluorescence staining for insulin (green) and glucagon (red) as well as nuclei (blue) visualized by DAPI are shown. Bars indicate 50 μm. *n*=9–10.

**Figure 6:**
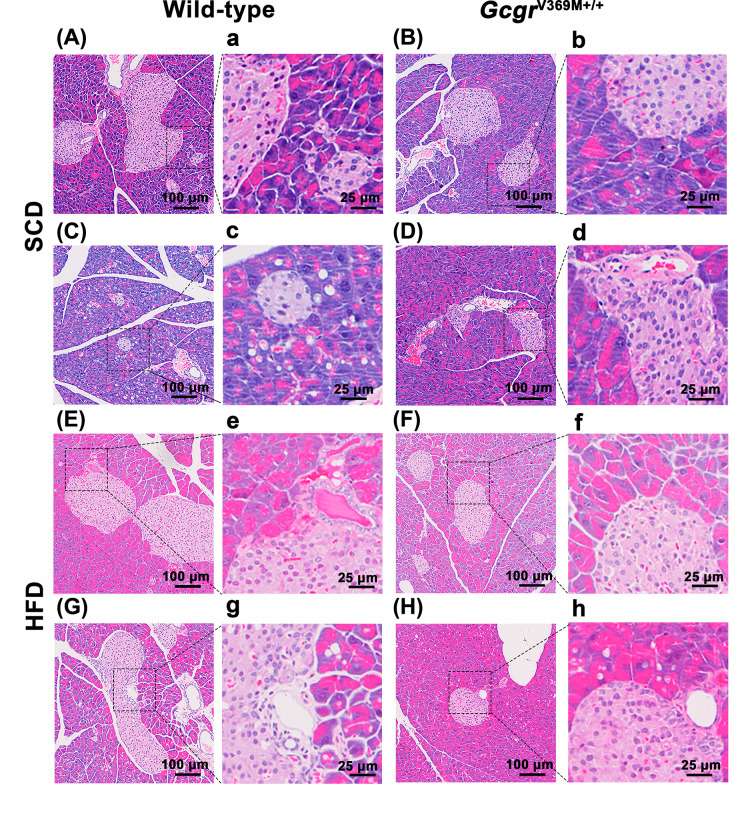
Pancreas histology of *Gcgr*^V369M+/+^ mice Microscopic evaluation (H&E staining) was conducted on 39-week-old male mice fed with SCD or HFD for 35 weeks. *n*=9–10. Scale bars are indicated in the slides. Images of low (100 μm) and high (25 μm) magnifications from two representative animals of each group are displayed. (**A, a, C, c**) WT mice fed with SCD. (**B, b, D, d**) *Gcgr*^V369M+/+^ mice fed with SCD. (**E, e, G, g**) WT mice fed with HFD. (**F, f, H, h**) *Gcgr*^V369M+/+^ mice fed with HFD.

As shown in Figure 5, *Gcgr*^V369M+/+^ mice demonstrated an overall α-cell hyperplasia, with elevated α-cell area and α-cell proliferation compared with WT mice on the same diet. However, pancreas histology examination did not see any notable difference between the two genotypes and no PNET was found at autopsy (Figure 6 and Table 3).

**Table 3:** Histological characteristics of the pancreas

Group			SCD		HFD	
			WT	*Gcgr*^V369M+/+^	WT	*Gcgr*^V369M+/+^
Number			9	9	10	9
Organ	Lesion	Degree	Lesion number			
Pancreas	Autophagic vacuoles	+	5	6	3	4
		++	1	0	3	3
	Interstitial monotype nucleocyte infiltration	+	1	2	7	5
		++	0	0	0	1
	Acinous cell atrophy	+	0	1	2	0

Pancreas histology examination was conducted in 39-week-old male mice fed with SCD or and HFD for 35 weeks. *n*=9–10. ‘+’ minor lesion; ‘++’ mild lesion; ‘+++’ moderate lesion; ‘++++’ severe lesions.

### Blood chemistry

While glucagon concentration of *Gcgr*^V369M+/+^ mice was higher than that of WT controls, insulin and GLP-1 levels remained similar (Table 4). It was noted that plasma BUN and CRE levels in *Gcgr*^V369M+/+^ mice on an SCD were significantly reduced whereas no such a difference was observed in *Gcgr*^V369M+/+^ mice receiving HFD, but their ALB level was significantly decreased.

**Table 4:** Plasma hormone levels and clinical chemistry

	SCD		HFD	
	WT	*Gcgr*^V369M+/+^	WT	*Gcgr*^V369M+/+^
Insulin (ng/ml)	1.33 ± 0.23	0.79 ± 0.14	4.47 ± 0.69	4.25 ± 1.34
Glucagon (pg/ml)	22.93 ± 2.85	127.20 ± 37.08**	57.42 ± 17.79	187.00 ± 42.31**
GLP-1 (pM)	11.16 ± 1.95	12.46 ± 1.84	10.13 ± 0.67	13.63 ± 3.71
ALB (mM)	26.99 ± 0.94	23.18 ± 1.55	25.75 ± 0.79	21.92 ± 1.23*
BUN (mM)	12.10 ± 0.47	9.87 ± 0.56**	9.67 ± 0.59	9.36 ± 0.33
CRE (mM)	6.96 ± 0.23	6.11 ± 0.30*	7.63 ± 0.28	6.54 ± 0.50

Plasma samples were collected on 39-week-old male mice fed with SCD or HFD for 35 weeks. GLP-1 measurement was carried out at week 38. Data shown are means ± SEM, *n*=9–10. *P<0.05 and **P<0.01 using Student’s *t* test and compared with WT mice on the same diet.

## Discussion

Glucagon has profound effects on glucose, amino acid and fatty acid metabolism that enable survival in conditions such as starvation and metabolic stress [5]. V368M (c.1102G > A, p.V368M), a naturally occurring GCGR single nucleotide polymorphism (SNP), was identified as a mild deleterious mutation leading to reduced ligand binding and down-regulation of glucagon signaling. Our previous studies confirmed that mice bearing homozygous V369M (c.1105G > A, p.V369M) substitution in the GCGR (*Gcgr*^V369M+/+^) display lower fasting blood glucose levels with improved glucose tolerance, hyperglucagonemia, pancreas enlargement and α-cell hyperplasia [22].

Since Mahvash disease is characteristic of hyperaminoacidemia, we determined plasma amino acid levels of both WT and *Gcgr*^V369M+/+^ mice fed with normal or high-fat diet. It was found that 3MHis and Cth levels were decreased but Ser and GABA level were increased in 35–39 weeks old *Gcgr*^V369M+/+^ mice on SCD. Excluding the influence of diets, V369M phenotypically elevated plasma concentrations of Asp, Trp, bAla, Hyp, Car, Ser, Glu, Hcit, bAib, Ans, Thr, Pro, Val, Lys, Cit, Asa, Ile, Ala, Arg, Aad, Orn and Met. It is well known that Ser, Ala, Pro, Arg, Val, Glu, Asp and Met are GAA, Lys is KAA, and Trp, Ile and Thr are G&KAA. They are regulated by glucagon to enhance glucose production [3]. Consistently, hepatic uptake accounts for approximately 70% of the TAA disposal and is greatest for GAA, such as Ala [24]. Mice with homozygous *Gcgr* deletion (*Gcgr^−/−^*) or treated with GRA1, a novel small molecule GCGR antagonist, exhibited practically hyperaminoacidemia, while Gln, Gly, Ala, Lys and Arg experienced the largest increase [7,9,11]. However, glucagon has no effect on BCAA [24,25], consistent with our observation.

Similar to what reported in the literature [25], V369M also increased the level of EAA in HFD-fed mice. Interestingly, these animals (35–39 weeks of age) presented lower amino acid levels than that of mice fed with SCD, which might have been caused by high insulin levels and impaired glucose tolerance in aging and diet-induced obesity (DIO) mice [26,27]. We also observed that *Gcgr*^V369M+/+^ mice fed with HFD tended to accumulate GAA, KAA and G&KAA in comparison with WT controls. Specifically, Ser, Ala, Pro, Arg, Met, His and Asn belonging to GAA were increased in *Gcgr*^V369M+/+^ mice; Thr and Tyr contributed to enhanced G&KAA levels and Lys elevated the level of KAA. Endogenous glucagon is lipolytic and ketogenic in humans while ketone bodies are produced by fatty acids and released by adipose tissue from KAA [28]. Mice with GCGR dysfunction could not dispose KAA well, resulting in KAA accumulation and lipid metabolic disorders. Nonetheless, the exact types of elevated amino acids are not identical among different laboratories. It is conceivable that in addition to amino acid turnover, aging, diet and fasting status prior to blood collection could all influence amino acid level detection.

Cth, a metabolic intermediate of Hcy to Cys, participating in the Met transmethylation cycle [29], was significantly reduced in *Gcgr*^V369M+/+^ mice on SCD. This suggests a barrier to transmethylation cycle. One can expect that altered Met transmethylation may impact hepatic very low-density lipoprotein (VLDL) export by changing the synthesis of phosphatidylcholine from pEtN, thereby contributing to the development of steatosis and steatohepatitis [30]. Our liver histological examination showed that three out of nine *Gcgr*^V369M+/+^ mice exhibited necrosis and steatosis, a phenomenon consistent with the reported observations in *Gcgr^−/−^* zebrafish [10] and in patients treated with LY2409021, a novel selective GCGR antagonist [31]. Furthermore, hepatic steatosis may impair glucagon-dependent enhancement of amino acid catabolism [32], as demonstrated by the three *Gcgr*^V369M+/+^ mice with liver steatosis and high amino acid levels. It is possible that a positive feedback between amino acid accumulation and hepatic steatosis may exist in *Gcgr*^V369M+/+^ mice. The products of protein metabolism are mainly BUN and CRE, which are excreted by the kidneys. *Gcgr*^V369M+/+^ mice showed low BUN and CRE levels compared with WT controls, suggesting a low rate of protein metabolism with enhanced kidney functions.

Inhibition of glucagon action leads to high concentrations of amino acids which act as sensors of hepatic glucagon signaling. Excessive glucagon secretion along with α-cell proliferation will follow to execute the functionality of the recently described liver–α-cell axis [7–9,13]. Especially, Gln regulates α-cell proliferation and mass via mTOR-dependent nutrient sensing [12,16], whereas Ala, Arg, Cys and Pro, but not Gln, are involved in the acute liver–α-cell axis of female mice [14]. Our previous and present results all demonstrated that excessive glucagon levels are accompanied by α-cell proliferation, and this phenomenon is linked with changes in amino acid levels as well as normal insulin and GLP-1 levels in *Gcgr*^V369M+/+^ mice. There was no abnormal islet function and PNET.

Hereditary, biallelic inactivating mutations of the *GCGR* gene is clinically characterized by hyperglucagonemia without glucagonoma, hyperaminoacidemia, transformation of hyperplastic islets into glucagon producing microadenomas and PNET, also known as Mahvash disease [17–20,33–35]. So far, only 11 cases have been described in the past 14 years due to a very limited incidence. V368M is a mild inactivating mutation, which may be valuable to study the onset and symptoms of Mahvash disease and the etiology of PNET [36]. Clearly, hyperaminoacidemia is capable of inducing hyperglucagonemia and α-cell hyperplasia, thereby activating the putative feedback loop, i.e. liver–α-cell axis. Further investigations are required to substantiate its existence with pathophysiological relevance.

## Supplementary Material

Supplementary Figures S1-S4

## Data Availability

The datasets generated during and/or analyzed during the current study are available from the corresponding authors on reasonable request.

## Abbreviations

ALB: albumin
BCAA: branched-chain amino acid
BUN: blood urea nitrogen
CRE: creatinine
EAA: essential amino acid
G&KAA: glucogenic and ketogenic amino acid
GAA: glucogenic amino acid
GCGR: glucagon receptor
GLP-1: glucagon-like peptide-1
HFD: high-fat diet
KAA: ketogenic amino acid
NEAA: nonessential amino acid
PNET: pancreatic neuroendocrine tumor
SCD: standard chow diet
TAA: total α-amino acid
WT: wildtype
