# Supplementary material for: Deleterious mutation V369M in the mouse GCGR gene causes abnormal plasma amino acid levels indicative of a possible liver–α-cell axis
Source: Biosci Rep. 2021 Jun 2;41(6):BSR20210758. doi: 10.1042/BSR20210758 (PMC8173527; doi:10.1042/BSR20210758)
Supplement: Supplementary Figures S1-S4 [file BSR-2021-0758_supp.pdf]

Supplementary Information

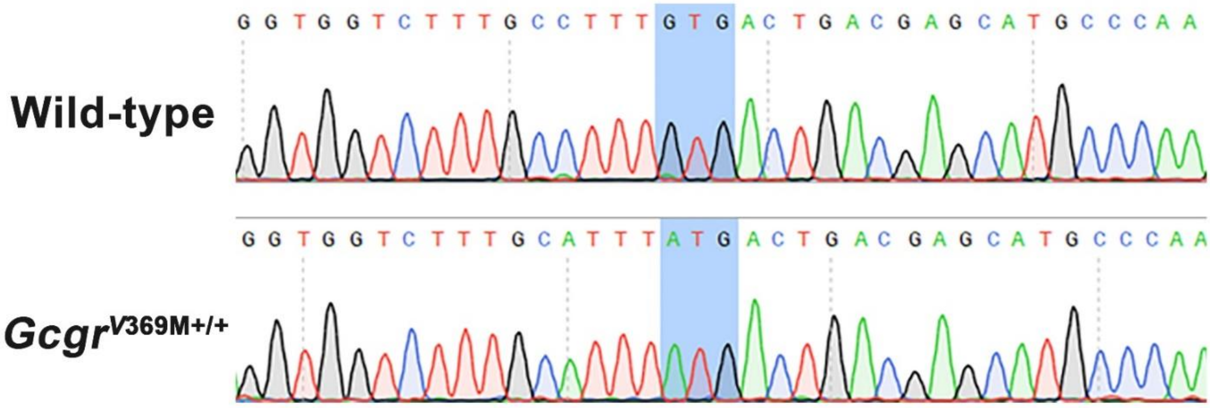

Supplementary Figure S1. Genotypes of wild-type and *Gcgr*$^{\text{V369M+/+}}$ mice by DNA sequencing.

A

Product #D12450J	gm%	kcal%
Protein	19.2	20
Carbohydrate	67.3	70
Fat	4.3	10
Total		100
kcal/gm	3.85	

Ingredient	gm	kcal
Casein, 30 Mesh	200	800
L-Cystine	3	12
Corn Starch	506.2	2024.8
Maltodextrin 10	125	500
Sucrose	68.8	275.2
Cellulose, BW200	50	0
Soybean Oil	25	225
Lard*	20	180
Mineral Mix S10026	10	0
DiCalcium Phosphate	13	0
Calcium Carbonate	5.5	0
Potassium Citrate, 1 H2O	16.5	0
Vitamin Mix V10001	10	40
Choline Bitartrate	2	0
FD&C Yellow Dye #5	0.04	0
FD&C Blue Dye #1	0.01	0

B

Product #D12492	gm%	kcal%
Protein	26.2	20
Carbohydrate	26.3	20
Fat	34.9	60
Total		100
kcal/gm	5.24	

Ingredient	gm	kcal
Casein, 30 Mesh	200	800
L-Cystine	3	12
Corn Starch	0	0
Maltodextrin 10	125	500
Sucrose	68.8	275.2
Cellulose, BW200	50	0
Soybean Oil	25	225
Lard*	245	2205
Mineral Mix S10026	10	0
DiCalcium Phosphate	13	0
Calcium Carbonate	5.5	0
Potassium Citrate, 1 H2O	16.5	0
Vitamin Mix V10001	10	40
Choline Bitartrate	2	0
FD&C Blue Dye #1	0.05	0

Supplementary Figure S2. Recipes of the standard chow diet (SCD) and high fat diet (HFD) used in this study.

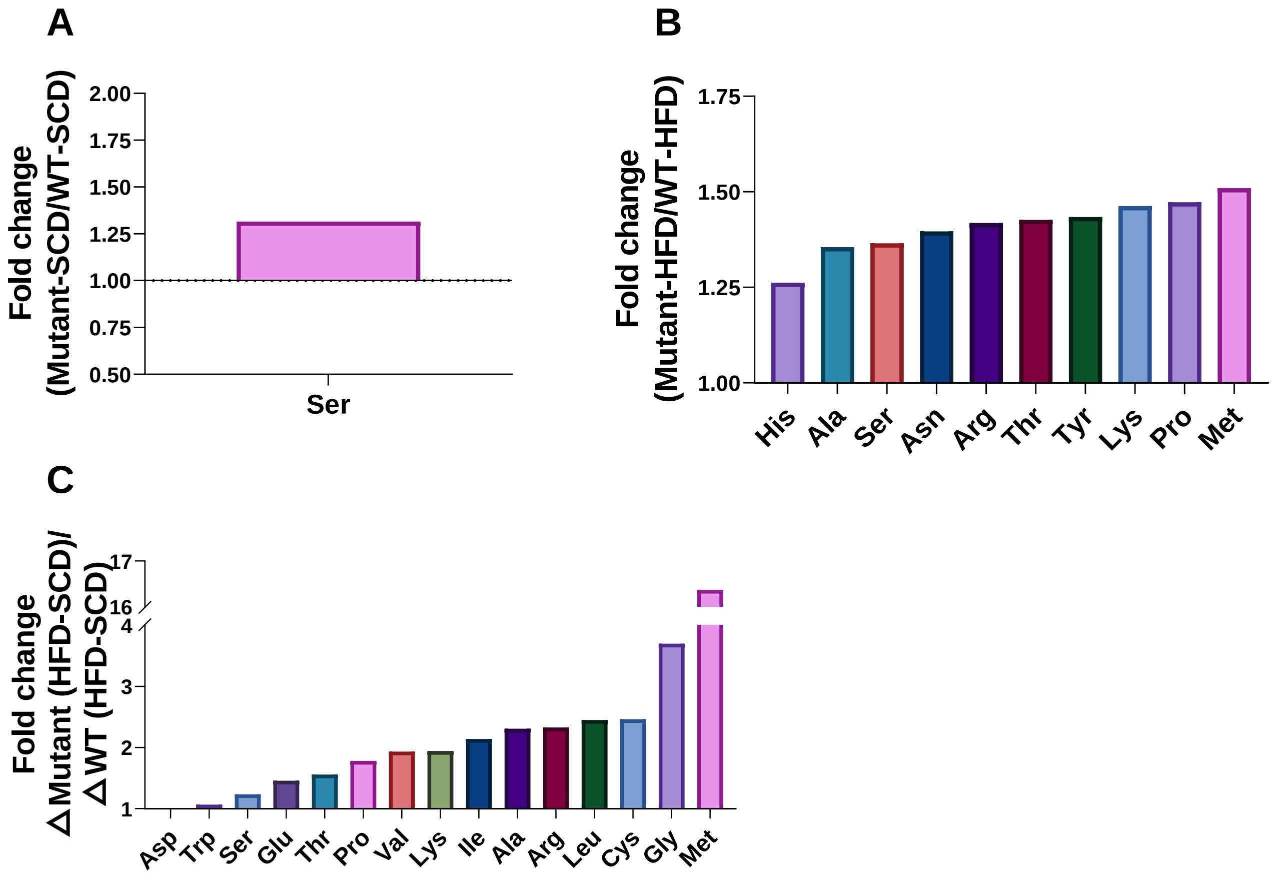

Supplementary Figure S3. Fold change of plasma amino acid levels. A, *Gcgr*$^{V369M+/+}$ (Mutant) mice displayed significantly different amino acids levels compared with wild-type (WT) mice in SCD group. B, *Gcgr*$^{V369M+/+}$ mice exhibited significantly elevated amino acid levels compared with that of WT in HFD group. C, The increased amino acid levels in *Gcgr*$^{V369M+/+}$ mice after excluding the effects of diet; Fold change = ($C_{Mutant\text{-}HFD}$ - $C_{Mutant\text{-}SCD}$)/($C_{WT\text{-}HFD}$ - $C_{WT\text{-}SCD}$). Plasma amino acid concentrations were measured in 35-39 weeks old male mice fed with SCD or HFD for 23-35 weeks. n = 15-17. SCD, standard chow diet; HFD, high fat diet.

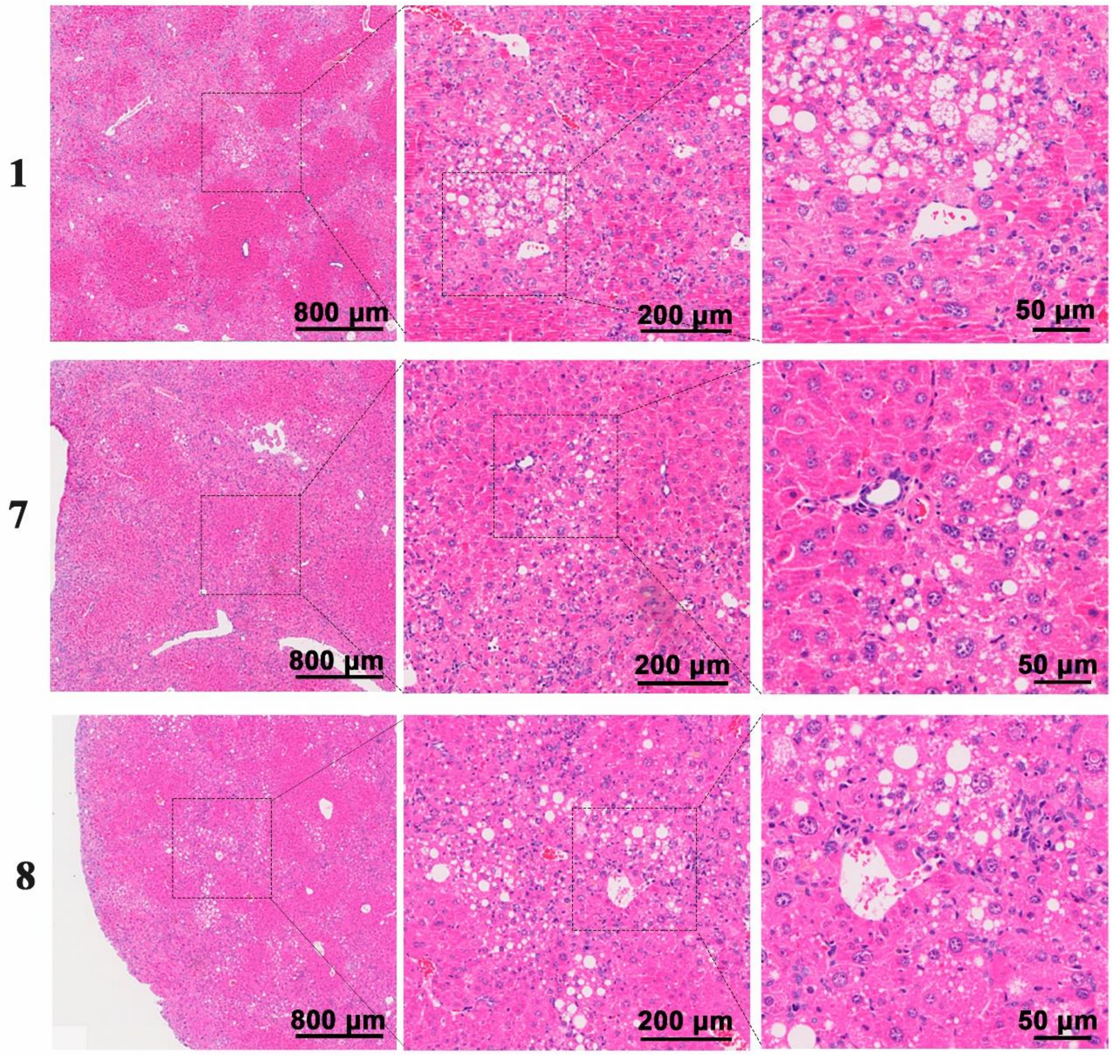

Supplementary Figure S4. Liver histology of *Gcgr*$^{V369M+/+}$ mice (Nos. 1, 7 and 8). Microscopy evaluation (H&E staining) was conducted on 39-week old male mice fed with SCD. Scale bars are indicated in the slides.
